# Age and altitude of residence determine anemia prevalence in Peruvian 6 to 35 months old children

**DOI:** 10.1371/journal.pone.0226846

**Published:** 2020-01-15

**Authors:** Roberto Alfonso Accinelli, Juan Alonso Leon-Abarca

**Affiliations:** 1 Instituto de Investigaciones de la Altura, Universidad Peruana Cayetano Heredia, Lima, Perú; 2 Facultad de Medicina Alberto Hurtado, Universidad Peruana Cayetano Heredia, Lima, Perú; 3 Hospital Cayetano Heredia, Lima, Perú; Vanderbilt University Medical Center, UNITED STATES

## Abstract

**Background:**

A Demographic and Family Health Survey (ENDES, for Encuesta Demográfica y de Salud Familiar in Spanish) is carried out annually in Peru. Based on it, the anemia prevalence was 43.6% in 2016 and 43.8% in 2017 using the WHO cutoff value of 11 g/dL and the altitude-correction equation.

**Objective:**

To assess factors contributing to anemia and to determine its prevalence in Peruvian children 6 to 35 months old.

**Methods:**

We used the MEASURE DHS-based ENDES survey to obtain representative data for11364 children from 6 to 35 months old on hemoglobin and health determinants. To evaluate normal hemoglobin levels, we used the original WHO criterion of the 5^th^ percentile in children without chronic malnutrition and then applied it to the overall population. Relationships between hemoglobin and altitude levels, usage of cleaning methods to sanitize water safe to drink, usage of solid fuels and poverty status were tested using methodology for complex survey data. Percentile curves were made for altitude intervals by plotting hemoglobin compared to age. The new anemia rates are presented in graphs by Peruvian political regions according to the degree of public health significance.

**Results:**

Hemoglobin increased as age and altitude of residence increased. Using the 5^th^ percentile, anemia prevalence was 7.3% in 2016 and 2017. Children from low altitudes had higher anemia prevalence (8.5%) than those from high altitudes (1.2%, p<0.0001). In the rainforest area of Peru, anemia prevalence was highest (13.5%), while in the highlands it was lowest (3.3%, p<0.0001). With access to safe drinking water and without chronic malnutrition, anemia rates could be reduced in the rainforest by 45% and 33%, respectively.

**Conclusion:**

Anemia prevalence in Peruvian children from 6 to 35 months old was 7.3% in 2016 and 2017.

## Introduction

In 1959 the World Health Organization (WHO) issued the first guidelines for anemia, defining it as hemoglobin (Hb) levels under 10.8–11.5 g/dL for 0.6 to 4-year-old children without acknowledging the arbitrariness of such values [1]. These guidelines were updated in 1968 to 11 g/dL for six-month-old to 6-year-old children, which is currently used [2].

Anemia remains a major health concern among young children living in developing countries. The high anemia prevalence has been related, among other factors, to iron deficiency, malnutrition [3], poverty, use of solid fuels, and absence of safe drinking water [4]. In an effort to reduce the anemia prevalence, many countries implemented programs to improve these conditions. While seemingly trivial, determining the factors contributing to anemia, and assessing the success of any preventive program depends on the ability to diagnose anemia itself. Unfortunately, defining anemia in countries with a significant population living at high altitude is not a straightforward task. The Andes mountains are the world's longest mountain range and boast some of the highest peaks. Stretching over 4,500 miles, the Andes cover seven countries—Venezuela, Colombia, Ecuador, Peru, Bolivia, Chile, and Argentina. In Peru, e.g., 27.3% of children under five years old reside at 2500 meters [5].These inhabitants have responded to chronic hypoxic conditions with increased levels of hemoglobin. Thus, a correction formula has been implemented to more precisely assess hemoglobin levels for children residing at high altitude as compared with those living at sea level. Intended to establish an easy and unique way to diagnose anemia while avoiding the altitude variability factor[6,7], this correction factor has also been adopted by the Peruvian guidelines which follow the World Health Organization (WHO) standards. This correction factor has never been critically evaluated. It is surprising, since all prevention strategies and measures of success ultimately rely on this factor.

Hemoglobin levels rise proportionately to the altitude of residence due to the effects of hypobaric hypoxia. Therefore, the WHO recommends correcting Hb values for people living above 1000 meters to obtain the equivalent Hb values for those living at sea level. This correction was determined in a study with a population of children older than 12 months who lived at an altitude of 3320 meters [8].

Because iron-deficient anemia has several negative effects on health and more importantly on the development of the nervous system [9], Peruvian children at risk receive daily micronutrients that include 12.5 mg of elemental iron [10]. Despite government efforts and a sustained increase in the country's Gross Domestic Product (GDP) since 1999 [11], anemia prevalence among children between 6 and 35 months old was 43.8% in 2017 and 43.6% in 2016. Puno, with most of its population around Lake Titicaca at 3848 meters (m), is the region with the highest anemia prevalence at 75.9%. However, chronic malnutrition rates there have decreased from 28% (2008) to 13% (2016) [12]. This poses a country-level paradox, how and why two programs that aim to supplement and improve the living standard in the same population show anemia rates in children from 6 to 35 months old that have failed to fall and have stalled above 40% in the last two decades.

Under the WHO guidelines, 45.2% of Bolivian children under five years old are anemic, with only 11.8% of cases consisting of iron-deficiency anemia. The study authors hypothesized that an erroneous altitude correction for Hb concentration or other causes of anemia were responsible for the high rates of anemia rather than iron deficiency [13]. In Peruvian pregnant teenagers living under 1000 meters (masl) anemia rates stand at 20.7%. However, at higher altitudes this rate doubles (e.g., Huancavelica at 3600 m with 48.3% and Puno at 3848 m with 45.6%) [14]. Researchers assumed that the high proportion of the indigenous population, with a different language and cultural beliefs, explained this difference, ever though teenage pregnant women living in these regions have higher percentages of iron supplement intake[15,16].

Gonzales suggested that the WHO guidelines for correcting Hb values at altitude have led to an overestimation in the prevalence of anemia in Peru [17]. In the Andean population, it favored an increase in the diagnosis of anemia and a decrease in the prevalence of excessive erythrocytosis [18]. When he applied the WHO criteria to infants from Puno with adequate iron reserves, anemia prevalence increased from 11.3% to 94.7%. In Ethiopia, for example, applying the WHO guidelines to anemic men and women living at 3700 masl, an increase in anemia was reported from zero to 28.3% and 48.5% respectively [19].

It is necessary to use age-specific criteria to diagnose anemia in children because hematological levels increase as children grow. The WHO Hb cutoff of 11 g/dL[2] for anemia was decided using the Second National Health and Nutrition Examination Survey (NHANES II, 1976 to 1980). This survey estimated normal hemoglobin values by age, with a cutoff for children from 12 to 35 months old at 10.7 g/dL and from 36 to 59 months old at 10.9 g/dL, not including children between 6 and 11 months old. In contrast, ENDES, the Peruvian Demographic and Family Health Survey (*Encuesta Demográfica y de Salud Familiar*, *ENDES*, *by its acronym* in Spanish) did include 6 to 11 months old group [20].

Finally, the WHO indicated that in people of African extraction, irrespective of age, the Hb anemia cutoff must be adjusted downwards by 1 g/dL [21], but in the study that was the basis for this correction (NHANES II), the difference found in African-American was 0.8 g/dL in children and 0.3 for adults [22]. Other than African Americans, ethnicity is not considered in the WHO cutoff anemia level of 11 g/dL. In Peru, people have been living at high altitudes in the Andean regions for at least ten thousand years, showing an array of genetic adaptations, but no adjustment for indigenous ancestry has been set to date [23].

Here we intend to determine the normal hemoglobin levels in children living in high altitudes and to introduce a better approximation for anemia cutoff values in Peruvian children from 6 to 35 months old, using ENDES information. This study is timely since information doesn’t currently exist describing hemoglobin (Hb) levels in young children that live at altitude. We will associate Hb levels with socio-demographic and altitude data to identify the most critical factors for determining hemoglobin levels in young children in Peru. The Hb distribution of healthy Peruvian children according to altitude and age is calculated, and follows the original suggestion of the WHO; namely that all children below the 5th percentile (p5) will be considered anemic[2].

## Methods

The Peruvian Demographic and Family Health Survey (*Encuesta Demográfica y de Salud Familiar*, *ENDES*, *for its acronym* in Spanish) collects nationally representative data on several health and socioeconomic factors yearly. Its methodology follows the recommended guidelines provided by the Monitoring and Evaluation to Assess and Use Results Demographic and Health Surveys (MEASURE DHS) program[20]. To select the year to analyze, the approach of the DHS program for Hemocue®-based blood samples is used to identify the years that had an SD less than 1.1 or above 1.5g/dL (S1 Table). We used the latest available version (2017) which surveyed 35,900 households to obtain data on children between 6 to 35 months old. Data included factors on age (months), sex, altitude of residence (meters, masl), height (meters), chronic protein-calorie malnutrition status, weight (in grams), hemoglobin (Hb, in g/dL), anemia status, poverty status (reported by quintiles and converted to dichotomous variables), usage of methods to access clean water, and use of solid fuels. After excluding records with invalid or missing hemoglobin data, defined by ENDES according to DHS methods [24], 11,364 children were included in the study.

For comparison purposes, anemia was defined using the original WHO anemia definition as the cutoff value at which more than 95% of healthy individuals have higher Hb levels [2] (5th percentile or p5) and the altitude-corrected hemoglobin levels, set at under 11 g/dL, using the CDC formula (Hb_corrected_ = Hb_measured_−Altitude adjustment; Altitude adjustment = -0.032*MASL + 0.022*MASL^2^) [7,25]. We excluded in this study all the children with Hb values <2.5g/dL or >20.0 g/dL as done before by a previous WHO report on anemia [26].

Because normal Hb levels are recommended to be evaluated on healthy individuals, the standard Waterlow classification[27–29] was used to exclude children with chronic malnutrition, defined as <95% of the expected height-for-age. For the purposes of this paper, “healthy children” is used as a synonym for children without chronic malnutrition. We then applied the Hb percentiles in the overall population to estimate new anemia rates.

Children were classified by age as 6 to 23 months old and 24 to 35 months old given the previously identified change in Hb values at the two-year-old point[6]. Linear regression with subpopulation analysis was used according to the survey data design to determine the relationship between several variables and Hb levels. Solid fuel usage was determined as the percentage of people using coal, lignite, charcoal, wood, straw, shrubs, grass, agricultural crop or animal dung for cooking or heating. Poverty status was defined as the first two categories (poorest and poorer) of the wealth index [30], which is a composite measure of a household's cumulative living standard. Usage of methods for access to clean water was defined as the percentage of people that apply any method to make water safe to drink-such as boiling, addition of chlorine or usage of filters. Altitude was classified as low (0 to 1524 m), moderate (1524 to 2438 m), high (2438 to 3657 m) and very high (3657 to 5486 m) because arterial blood saturation declines as altitude increases, and these cutoff points discriminate the changes of human physiological response to altitude [31,32].

Percentile graphs were obtained using the Harrell-Davis distribution-free quantile estimator and then smoothed using quadratic regression lines [33]. The degree of public health significance [34] for anemia rates is presented in various graphs by Peruvian political regions. Additional analysis by Peruvian natural regions Costa (Coast, mostly lowlands), Sierra (the Andean highlands) and Selva (the rainforest)) for anemia rates and associated factors such as solid fuel exposure, access to safe drinking water and the presence of chronic malnutrition is presented as supplementary data. Weights, strata, and primary sampling units were used according to the Demographic and Health Surveys (DHS) design of complex surveys to preserve national-level representativeness of data. The STATA 15 software was used to analyze the data, and p<0.05 was considered as the statistical significance reference value. The Bonferroni correction was applied, as necessary, to make corrections for the number of tests performed.

### Ethics statement

Cayetano Heredia’s University Ethics Committee approved this research with registry numbers 103317 and 103318. This study used INEI’s ENDES anonymized secondary data. Participation in this survey required a written informed consent.

### Data sharing statement

Materials used in this study are publicly available at Instituto Nacional de Estadística e Informática del Perú (INEI) webpage: http://iinei.inei.gob.pe/microdatos/

## Results

Among children aged 6 to 35 months old, 27.2% show chronic malnutrition, 34.8% live in houses where solid fuels were used for cooking, 48.9% live in poverty, and 89.4% have access to safe drinking water. Most of the children tested live at low altitudes (69.7%) and were 6 to 23 months old (60.5%) (Table 1).

**Table 1:** Distribution of children allocation and mean hemoglobin differences by variables.

Variables		% (95% CI)	Hb (g/dL)				
			No	n	Yes	n	p
Other variables	Solid fuel exposure	34.8% ± 1.7	11.6 ± 0.04	6906	11.9 ± 0.1	4371	**<0.0001**
	Chronic malnutrition	27.2% ± 1.3	11.6 ± 0.04	6120	11.8 ± 0.1	5216	**<0.01**
	Clean water measures	89.4% ± 0.9	11.3 ± 0.1	1246	11.7 ± 0.04	10118	**<0.0001**
	Poverty	48.9% ± 1.8	11.6 ± 0.1	5109	11.8 ± 0.1	6255	**<0.0001**
Sex	Male	50.04% ± 1.3	11.6 ± 0.1 11.7 ± 0.1		5771 5593		**<0.0001**
	Female	49.96% ± 1.3					
Age (mo)	6–23	60.5% ± 1.2	11.4 ± 0.1 12.1 ± 0.1		6905 4459		**<0.0001**
	24–35	39.5% ± 1.2					
Altitude	Low (0–1524)	69.7% ± 1.6	11.2 ± 0.04 12.2 ± 0.1 12.9 ± 0.1 13.5 ± 0.1		7398 855 2220 891		**<0.0001**
	Moderate (1524 to 2438)	6.5% ± 1.0					
	High (2438–3657)	17.3% ± 1.6					
	Very high (3657–5486)	6.6% ± 0.9					

Percentages and unadjusted means are estimated based on DHS methodology. Total n = 11364, except where missing and invalid values existed as coded in the original database. Hb = hemoglobin, mo = months

Bivariate (uncorrected) analysis showed that Hb was higher in girls than boys (11.7 vs. 11.6g/dL) and in older children versus younger children (12.1 vs. 11.4g/dL) (Table 2). Those who were exposed to solid fuels had higher hemoglobin values (11.6 vs. 11.9 g/dL, p<0.0001) (Table 1) and the percentage of children living in these conditions increased with altitude; namely 24.1% at low altitude and rising to 63.5% at high altitude (Table 2). Children with chronic malnutrition had higher Hb levels (Table 1), and the percentage of children with chronic malnutrition increased with the altitude of residence, being more than double (46.7%) in those at very high altitude compared with those from low altitude (21.5%) (Table 2). Higher Hb values were seen in children living in poverty and those at very high altitudes doubled up (82.3%) compared to those living at low altitudes (38.5%) (Table 2).

**Table 2:** Data distribution using altitude thresholds.

Altitudes	n	Solid fuel exposure	N	Chronic malnutrition	n	Clean water measures	N	Poverty	n	6–23 months	n	24–35 months	n
Low	7398	24.1% ± 1.8	2096	21.5% ± 1.4	1654	89.2% ± 1.1	6428	38.5% ± 2.2	3390	60.4% ± 1.5	4467	39.6% ± 1.5	2931
Moderate	855	44.6% ± 6.3	365	30.0% ± 4.8	239	86.9% ± 4.0	773	57.7% ± 6.0	492	63.1% ± 3.4	531	36.9% ± 3.6	324
High	2220	63.5% ± 3.9	1400	41.7% ± 2.8	888	90.3% ± 2.1	2076	75.1% ± 3.2	1676	59.9% ± 2.4	1361	40.1% ± 2.4	859
Very high	891	62.6% ± 5.7	510	46.7% ± 5.1	434	91.1% ± 2.4	841	82.3% ± 4.0	697	60.8% ± 3.6	546	39.2% ± 3.7	345

Percentages shown represent children affected by each variable in each category along with the number of observations used.

Hb increased with altitude rising from 11.2 g/dL for those living at low altitudes to 13.5 g/dL for those living at very high altitudes, with the hemoglobin levels being significantly different among the four altitude levels (Table 1). At low altitude, children Hb levels were negatively associated with solid fuel exposure, chronic malnutrition, and poverty, and positively affected by clean water, age, and sex. At high or very high altitudes, age was the sole factor associated with Hb levels. However, at moderate altitude (1524 to 2438 m) gender was a positively related factor, while poverty and its interaction with solid fuels were negatively associated (Table 3).

**Table 3:** Linear regressions of hemoglobin (g/dL) by altitude.

Variables	Low altitude (0–1524 m)		Moderate altitude (1524 to 2438 m)		High altitude (2438–3657 m)		Very high altitude (3657–5486 m)	
	Coefficient	p	Coefficient	P	Coefficient	p	Coefficient	P
Solid fuel exposure	-0.13	**0.015**	0.06	0.608	-0.11	0.129	-0.01	0.95
Chronic malnutrition	-0.12	**0.041**	-0.02	0.892	0.05	0.407	0.09	0.517
Clean water	0.33	**<0.0001**	-0.04	0.792	0.05	0.656	-0.05	0.73
Poverty	-0.27	**<0.0001**	-0.52	**<0.0001**	0.01	0.894	0.05	0.738
Age*	0.61	**<0.0001**	0.7	**<0.0001**	0.6	**<0.0001**	0.57	**<0.0001**
Sex**	0.11	**0.001**	0.19	**<0.0001**	0.16	0.003	0.15	0.13
Interaction: Poverty and solid fuel use	-0.001	0.996	-0.53	**0.04**	0.19	0.183	-0.15	0.562
Interaction: Poverty and chronic malnutrition	0.01	0.882	0.33	0.148	-0.11	0.46	0.06	0.793
Constant term⁺	10.01	**<0.0001**	11.26	**<0.0001**	11.79	**<0.0001**	12.5	**<0.0001**

Each model has a p<0.0001.

*6–23 months vs 24–35 months.

**Male vs female.

^+^Expected hemoglobin value when all other variables = 0

**Fig 1:**
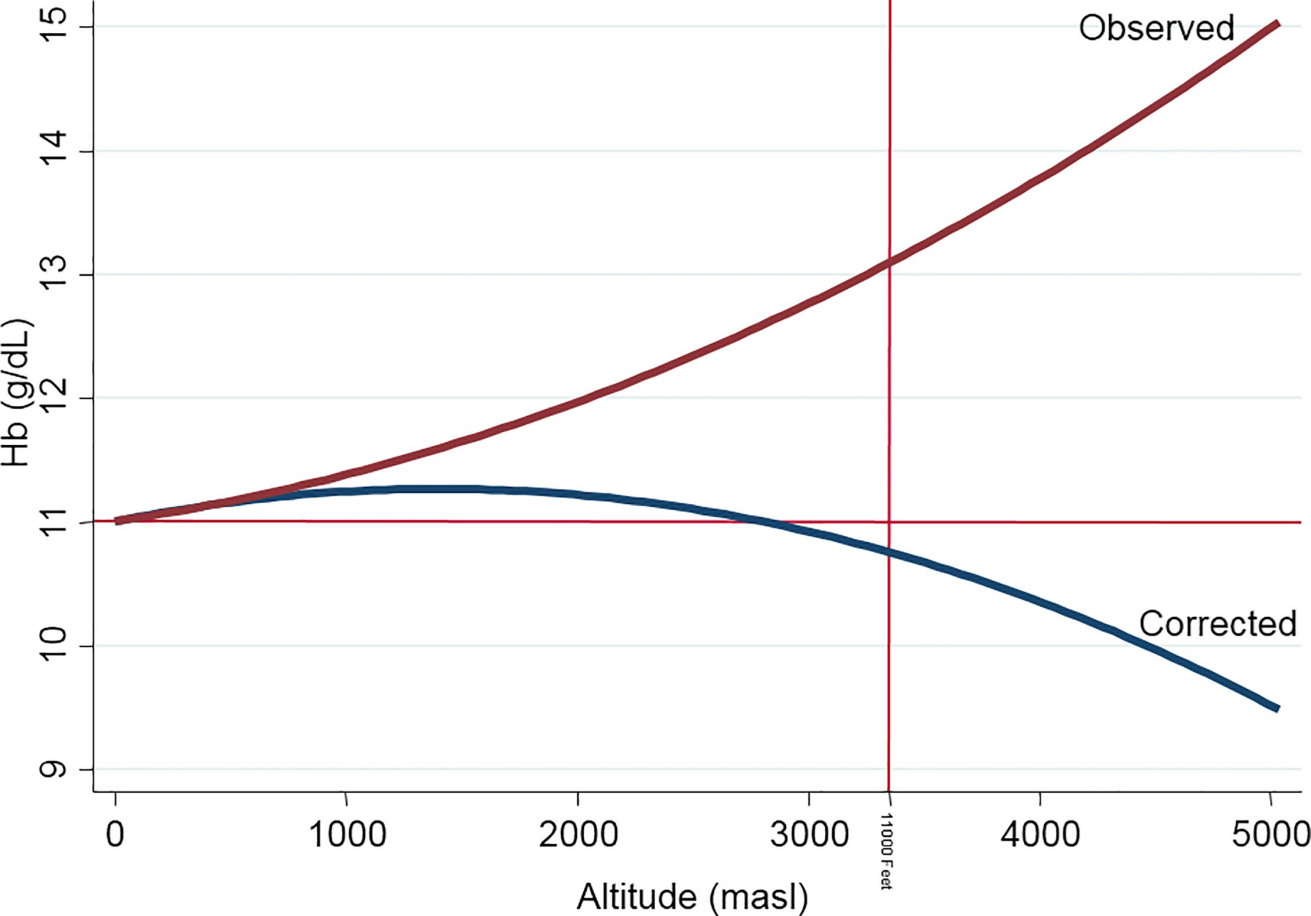
Hemoglobin trends according to observed and WHO altitude-corrected values. Red line parallel to X axis represents the 11 g/dL anemia threshold and the red line parallel to Y axis the 11000 feet boundary of WHO/CDC altitude-correction factor equation.

In Fig 1, we plotted Hb (g/dL) with residence at elevations above sea level (m). The bold red line represents the observed Hb average that follows a quadratic pattern with positive concavity. We also show in a blue line for the WHO correction Hb average, which also has a quadratic pattern but with negative concavity.

**Fig 2:**
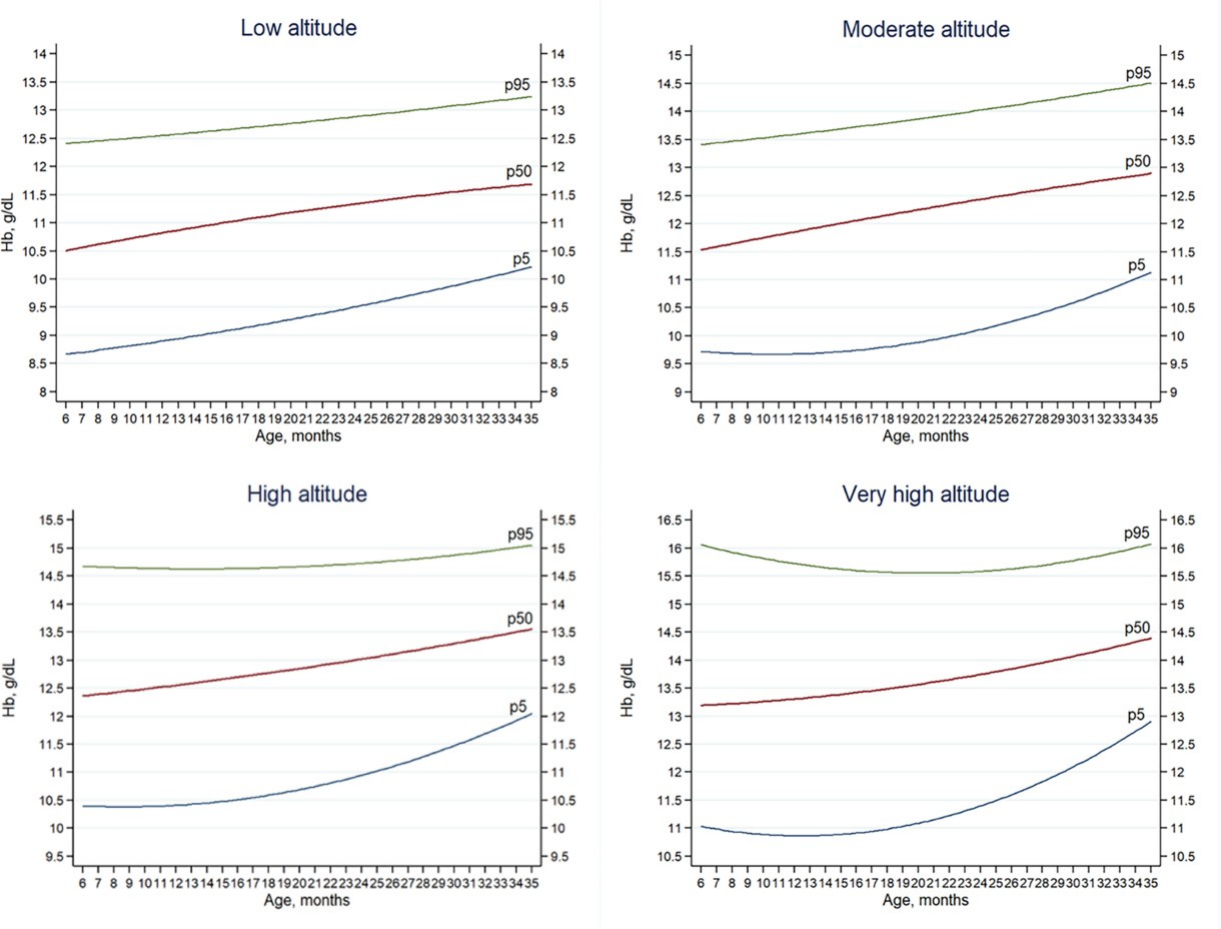
Hemoglobin percentiles by age and altitude categories (masl). In order, from bottom to top: p5 (blue), p50 (red), p95 (green). Percentile curves as estimated using altitude of residence show that Hb increases along with age. However, some differences arise: At lower altitudes, the p5 curve follows the equation 8.47+ 2.87*10^−2^*x+ 6.04*10^−4^*x^2^ and p50 follows the equation 10.16+ 6.12*10^−2*^x- 5.02*10^−4^*x^2^. At moderate altitudes the p5 curve follows the equation 9.94–5.17*10^−2^*x+ 2.45*10^−3^*x^2^ with the lowest estimated value found at 10.6 months, and the p50 curve follows the equation 11.20+ 5.81*10^−2^*x- 2.72*10^−5^*x^2^ which shows an upward trend across the age range. At high altitudes the p5 curve follows the equation 10.55–4.08*10^−2^*x+ 2.38*10^−3^*x^2^ with the lowest estimated value found at 8.6 months. The p50 equation is 12.20+ 2.42*10^−2^*x+ 4.11*10^−4^*x^2^, which follows an upward curve for the age range. At very high altitudes p5 follows 11.49–10.09*10^−2^*x+ 4*10^−3^*x^2^ with the lowest estimated value found at 12.5 months. The p50 follows 13.15+ 1.31*10^−3^*x+ 9.78*10^−4^*x^2^ which has an upward trend for the given age range.

Hb averages by age are shown in Tables 4 and 5, along with the estimated prevalence of both conditions when applied to the general population. The highest Hb means are found in children aged 24–35 months old living at very high altitude (14.0 g/dL) and high altitudes (13.3g/dL) while the lowest means were found in children living at low altitudes with 6–23 months old (11.0 g/dL) and 24–35 months old (11.5g/dL). Anemia rates (p5) differed (7.3% for 2017) from those obtained according to WHO guidelines (43.6% for 2016 and 2017). The highest rates are found in children between 24–35 months old living at low altitudes (11.4%). The lowest rates are found in children between 24–35 months old who live at high altitude (1.0%). (Table 3 and Fig 2)

**Table 4:** Hemoglobin (g/dL) in healthy children along estimated population anemia rates based on p5 (2017).

Altitude	Ages (mo)	n	Mean Hb*	SD	p5	Estimated prevalence	[95% CI]	
Low	6–23	3318	11	1.1	9	5.20%	4.40%	6.10%
	24–35	2416	11.6	0.9	10	14.30%	12.80%	16.00%
Moderate	6–23	364	11.9	1.2	9.8	6.30%	4.40%	8.90%
	24–35	252	12.7	1	11	11.40%	7.80%	16.50%
High	6–23	747	12.6	1.2	10.6	1.30%	0.70%	2.30%
	24–35	584	13.3	1	11.5	1.00%	0.40%	2.50%
Very high	6–23	242	13.2	1.4	10.5	5.00%	3.10%	7.80%
	24–35	215	13.9	1	12.4	7.40%	4.50%	12.00%
					Total	7.30%	6.60%	7.90%

Differences between age groups and altitude with a p<0.0001. Hb = hemoglobin, mo = months

**Table 5:** Hemoglobin (g/dL) in healthy children along estimated population anemia rates based on p5.

Altitude	Ages (mo)	n	Mean Hb*	SD	p5 (2016)	p5	2016	[95% CI]	
Low	6–23	2937	10.9	1.1	9	9	4.70%	4.00%	5.60%
	24–35	2216	11.6	1	10	10	13.90%	12.40%	15.60%
Moderate	6–23	337	12	1.2	10	9.8	4.40%	2.50%	7.60%
	24–35	269	12.7	1	11	11	9.20%	6.40%	13.10%
High	6–23	689	12.5	1.4	10.1	10.6	2.30%	1.50%	3.60%
	24–35	499	13.3	1	11.6	11.5	0.80%	0.30%	1.90%
Very high	6–23	216	13.2	1.4	10.8	10.5	5.80%	3.70%	9.10%
	24–35	181	14	1.1	12.1	12.4	9.10%	6.30%	13.00%
						Total	7.30%	6.70%	8.00%

Differences between age groups and altitude are significant at a p<0.0001 (2016 data, 11163 children from 6 to 35 months). 2017 p5 cutoffs used for percentage and CI estimation. Hb = hemoglobin, mo = months

**Fig 3:**
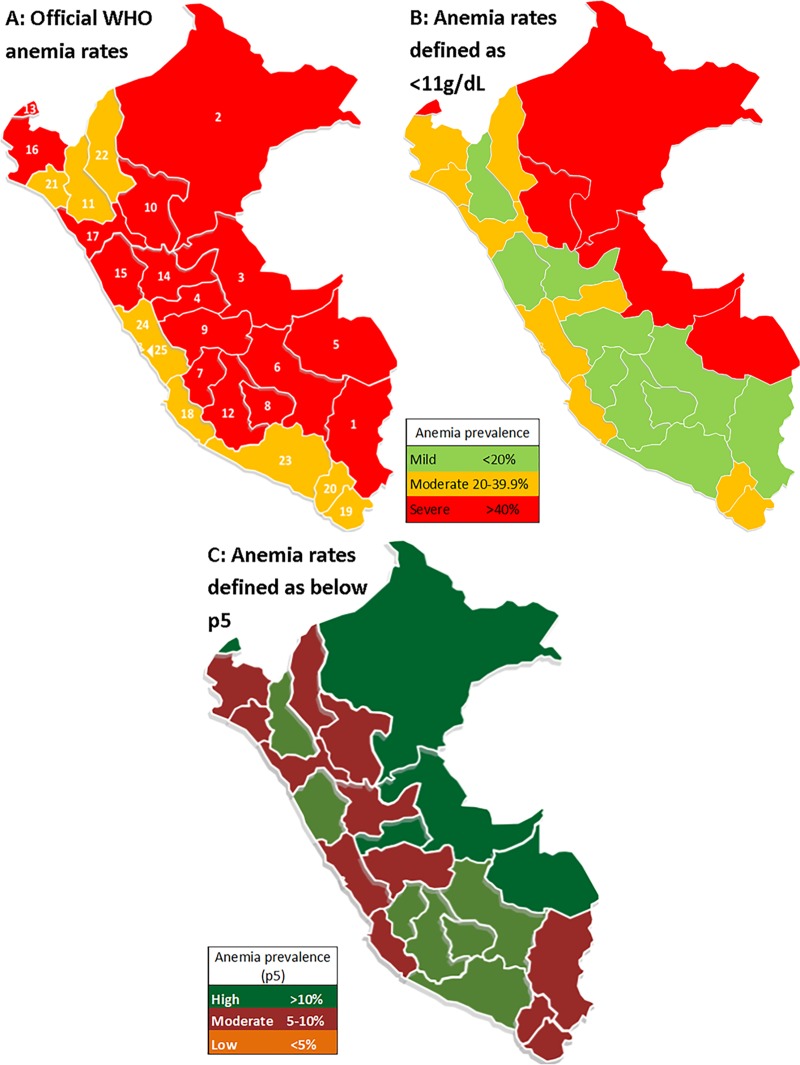
Anemia rates by political regions. (A) Anemia using WHO guidelines. The numbers correspond to political regions as presented in Table 4. (B) Anemia using Hb <11g/dl as cutoff point. (C) Recalculated anemia using p5.

In Table 6 and Fig 3, the 25 regions are shown along with their corresponding anemia rates for 2017 using the WHO definition, Hb below 11g/dL, and the p5 cutoffs obtained in the present study. The three regions with the highest anemia rates according to the WHO definition were Puno (75.6%), Loreto (61.5%) and Ucayali (59.2%), while the p5 cutoff showed Ucayali (15.5%), Loreto (12.5%), and Madre de Dios (11.7%) to be the highest regions. The three latter ones are regions in the rainforest of Peru, while Puno is mostly a highland region. Peruvian natural regions have different sets of environmental and socioeconomic factors, which may translate into different anemia rates in children. As seen in Fig 1, children who live at very high altitudes have high Hb levels. As such, the rainforest (low altitude) tends to have the highest anemia rates (WHO: 53.8%, p5:13.5%), while the highlands show a varying prevalence (WHO: 52.4%, p5: 3.3%). The WHO criteria does not detect differences in anemia rates between the highlands and rainforest (p>0.05), but p5 criteria do (p<0.0001). Moreover, the highest anemia rates tend to concentrate at very high altitudes according to the WHO criteria (70.5%, p<0.0001) while p5 shows the highest rates in low altitude children (8.8%, p<0.0001). (Table 7)

**Table 6:** Anemia rates by Peruvian political regions (2017), sorted by official anemia rates.

Political region		Natural region (survey data)	WHO definition	Hb with 11g/dl cutoff	p5 cutoff
1	Puno	H+R	75.60%	5.80%	8.00%
2	Loreto	R	61.50%	61.50%	16.00%
3	Ucayali	R	59.20%	59.20%	20.10%
4	Pasco	H+R	58.40%	25.10%	10.20%
5	Madre de Dios	R	57.60%	57.60%	12.30%
6	Cusco	H+R	56.60%	7.50%	3.10%
7	Huancavelica	C+H	55.10%	3.80%	2.10%
8	Apurimac	H	55.10%	4.30%	1.00%
9	Junin	H+R	53.90%	15.40%	5.40%
10	San Martin	R	51.30%	49.10%	8.50%
11	Amazonas	H+R	51.00%	38.00%	9.60%
12	Ayacucho	H+R	49.30%	10.90%	1.40%
13	Tumbes	C	46.70%	46.70%	12.00%
14	Huanuco	H+R	44.60%	17.50%	6.70%
15	**National average**	**C + H + R**	**43.80%**	**29.70%**	**7.30%**
16	Ancash	C+H	42.40%	17.00%	3.30%
17	Piura	C+H	41.70%	39.10%	8.10%
18	La Libertad	C+H	41.40%	26.00%	5.50%
19	Ica	H+R	39.80%	39.30%	6.70%
20	Tacna	C+H	38.00%	33.10%	6.80%
21	Moquegua	C+H	37.60%	27.60%	6.30%
22	Lambayeque	C+H	37.40%	36.50%	6.00%
23	Cajamarca	C+H+R	37.40%	12.80%	4.60%
24	Arequipa	C+H	34.90%	11.90%	3.90%
25	Lima	C+H	34.70%	34.30%	7.60%
26	Callao	C	32.80%	32.80%	7.60%

Natural regions: C = Coast, H = Highlands, R = Rain forest.

**Table 7:** Anemia rates by natural regions and altitude.

Anemia rates by		WHO	p5
Natural regions	Coast	36.1%ᵃ	5.3%ᵈ
	Highlands	52.4%ᵇ	3.3%ᵉ
	Rain forest	53.8%ᶜ	13.5%ᶠ
Altitude	Low	40.5%^g^	8.8%^h^
	Moderate	37.1%^i^	8.2%^j^
	High	49.6%^k^	1.2%^l^
	Very high	70.5%^m^	5.9%^n^

Linear combination of estimates p value: ab, ac, de, df, ef, gk, gm, ik, im, km, hl, jl, ln = <0.0001; bc = 0.395; gi = 0.804; hj = 0.597; hn = 0.036; jn = 0.72.

Anemia rates calculated with p5 are higher in the rainforest amongst children with chronic malnutrition and those whose parents do not report using any methods to get clean drinking water (p<0.05). Using the WHO definition, anemia rates in children exposed to solid fuels, with chronic malnutrition and not using any methods to get clean drinking water are higher (p<0.05), with the exception of children whose parents report using measures to clean water at highlands, who have similar values than those without them (52.5 vs 52.4%, p = 0.998). (S3 Table)

## Discussion

We found that 6 to 23 months old Peruvian children had an average Hb value of 11.4 ± 0.1 and at 24 to 35 months of age, 12.1 ± 0.1 g/dL (<0.0001). (Table 1) Using white children data from the Second National Health and Nutrition Examination Survey in the United States (USA, NHANES II), Dallman found 0.3 g/dL higher Hb value in 36 to 59 months than in 12 to 35 months old children [35]. For 6 to 60 months old children, the WHO Hb cutoff point for anemia is 11 g/dL, and it does not change across the 6–35 months age range [36]. Thus, the WHO cutoff identifies a higher prevalence of anemia in the youngest children (who are the ones with the lowest hemoglobin value). For example, in the case of Peru, 59.6% of those between 6 and 11 months are considered anemic, vs. 23.6% at 12 to 35 months of age[34]. To illustrate further using the database of NHANES II, Yip found 10.7 g/dL as the cutoff anemia point in children 12 to 35 months old and 10.9 g/dL for those 36 to 59 months old [37].

Therefore, if we use the WHO cutoff point of 11 g/dL in the same American children, the anemia prevalence would be overestimated. A similar situation would happen in undeveloped countries. In Rwanda, children less than five years old had an anemia prevalence of 30.9%. However, iron deficiency prevalence, defined by low serum ferritin, was 5.9% and by serum transferrin receptor was 3.1% [38]. Because 42% to 50% of anemia in children is expected to be caused by iron deficiency^4^[34], the prevalence of anemia in Rwanda would be overestimated in about two thirds of the children by using the WHO 11 gr/dL cutoff. In addition, use of the WHO 11 gr/dL cutoff criterion could explain why 88% of 6 to 30 months old anemic children from north India who received iron for two months remained anemic even after correction for iron deficiency [39].

The use of threshold values to classify anemia were first published in the report of 1958 WHO Study Group [2] and these were chosen arbitrarily. The WHO revision of 1968, for children between 6 to 59 months old, recommending 11 g/dL as the anemia cutoff level [2] was based on five research studies, one of these being an unpublished paper [40–43]. Moreover, none of these studies was conducted in a pediatric population. With a single cutoff point, and given the rise of Hb with age, the prevalence of anemia will always be higher in children younger than one-year and will decline as the child's age increases. This effect of age likely explains the high prevalence of anemia in children in Rwanda (2007–2008), which was 74.8% between 6 to 8 months, 69.8% between the age of 9 to 11 months, 53.4% from 12 to 17 months, 43.4% between 18 and 23 months, 36.6% between 24 and 35 months, 30.6% between 36 and 47 months, and 25.5% between 48 and 59 months of age [44].

Children with access to unsafe drinking water, or exposed to solid fuels, chronic malnutrition, or poverty would be expected to have lower values[45,46]. Since a higher frequency of acute diarrheal and parasitic diseases would be expected, which exposes these children to chronic inflammation and in some cases, gastrointestinal blood loss [47]. Also, at a global level, anemia rates in children are associated with the frequency of solid fuel use [48] given that traditional, inefficient stoves generate indoor pollution, higher amounts of particulate matters in the alveoli, where macrophages phagocytize them initiate an inflammatory response [49,50]. Yet, we found in Peru that children in poverty, exposed to solid fuels, or in chronic malnutrition had higher Hb values (Table 1). This apparent paradox can be explained insofar as 2.6 more Peruvian children are exposed to solid fuels (24.1% vs. 62.6%) at higher altitudes than lower altitudes, with 2.2 and 2.1 times as many sufferings from chronic malnutrition (21.5% vs. 46.7%) or poverty (38.5% vs. 82.3%, Table 2) respectively. Despite the influence of unsafe drinking water, malnutrition or poverty, high altitude children have, on average, 2.3 g/dL higher Hb levels (Table 1).

To evaluate anemia adequately in the population living at altitude, WHO proposed correcting Hb values according to altitude of residence. This correction equation was constructed for children older than 12 months living between 0 to 3352 m[7,8]. With the WHO Hb correction for altitude, the Hb-age curve for Peruvian children has a negative concavity and quadratic trajectory, which differed markedly from the Hb vs. altitude curve with positive concavity shown in Fig 1. When applying this correction to Puno (3848 m) in infants with adequate iron reserves, anemia prevalence increases from 11.3% to 94.7% [17]. Similarly, in healthy adults from Ethiopia (3700 m) with iron reserves above zero, anemia levels increased in men and women from zero to 28.3% and 48.5%, respectively [19]. Thus, the WHO correction overestimates anemia.

We have found in Peruvian children that Hb increases with altitude (Table 1). Only at low altitude are childhood Hb levels negatively associated with solid fuel exposure, chronic malnutrition, and poverty, and positively affected by clean water, age, and sex (Table 4). At moderate altitude (1524 to 2438 m) female gender is positively related to Hb, while poverty and its interaction with solid fuels are negatively associated. In addition, at high altitude, female gender is positively related to Hb. The linear regressions of hemoglobin (g/dL) by altitude demonstrate that age is solely associated with Hb levels (Table 3).

Therefore, our results suggest that Hb levels in children are associated with age and the altitude of residence. At altitudes above 1524 meters, hypobaric hypoxia is the strongest determinant of Hb levels [51]. Social conditions are associated with Hb values at sea level and up until 1524 m; at higher altitudes (over 2438 m) their effects seem to disappear. The reduction in anemia prevalence at high altitudes is most likely due to the effects of hypoxia on stimulating the production and release of erythropoietin (EPO), the most potent stimulator of erythropoiesis, in renal [52] and extra-renal tissues [53–62]. Anemia is a condition where the number of red blood cells or their oxygen-carrying capacity is insufficient to meet physiologic needs [63]. Its incidence varies according to age, sex, altitude of residence, smoking, and pregnancy status [64]. Iron-deficiency anemia in young children has detrimental effects on neurological development, cognitive function, exercise tolerance, immune function, and school performance [65,66].WHO defines anemia as a condition in which Hb concentration *is lower than normal* and *is diagnosed when the concentration of Hb falls below established cutoff values* [67] (the 5th percentile of those obtained of those healthy people of same sex, age, and pregnancy condition).

We found a sex difference of only 0.1 g/dL of Hb, being higher for girls (Table 1), and when altitude and age are considered, this difference disappears. Given the varying nature of sex and altitude on Hb levels, we consider it unsuitable to design a correction formula that combines age and residential altitude. Therefore, we used data from more than 11,000 Peruvian children in the ENDES survey aged 6 to 35 months screened for their Hb yearly to build four anemia cutoff curves, one for each altitude interval of residence relating Hb and age in months (Fig 2). Using the 5^th^ percentile as a cutoff value for defining anemia, we found an anemia prevalence of 7.3% in 2017 and 2016 (Tables 4 and 5). On the other hand, with the WHO cutoff point of 11 g/dL and use of the WHO high-altitude correction factor, the anemia rates in Peru for 2016 and 2017 were 43.6% and 43.8% respectively[20], over 6 times the prevalence calculated in the present study. More generally and at a global level, the use of the WHO age-independent cutoff is the reason that the highest anemia prevalence is found in preschool children[37], because the lowest Hb levels are at 6 to 11 months old and values increase with age[47,68,69] as shown in NHANES II and Peruvian population (Table 1). Henceforth, we recommend the use of age-specific criteria for the diagnosis of anemia.

It is possible to find population differences in Hb levels. The WHO Hb anemia cutoff guidelines add one g/dL to the value obtained for people for African ancestry regardless of age [70]. However, no other racial differences are taken into account even though different WHO thresholds are indicated for defining anemia in African Americans (-1.0 g/dl), Jamaican girls (-1.07 g/dL), Vietnamese (-1.0 g/dL), Greenland women (-0.6 g/dL) and Greenland men (-0.8 g/dL) [40]. Likewise, there is an Hb difference of 0.28 g/dL among white individuals with northern versus southern European ancestry [71]. People have been living at high altitudes, in the Andes, Tibet, and Ethiopia, for thousands of years [72]; the Tibetan and Ethiopian populations have resided at high altitudes for much longer [73]. At a similar altitude of residence, Andean people have higher Hb values; perhaps because Tibetan and Ethiopian populations have developed genetic adaptations affecting regulation of Hb levels [74]. However, Andean population also demonstrate albeit genetic adaptations affecting the cardiovascular [23] and other systems involved in the regulation of fetal growth and birth weight [75]. Having constructed our equations with the Peruvian children included in the ENDES survey, we have taken into consideration and incorporated genetic and other factors influenced by population differences in the regulation of Hb levels.

At a regional level, Puno (3848 m) has the highest WHO-defined anemia prevalence (75.6%) (Fig 3). With a WHO cutoff of 11 g/dL, but without the WHO-altitude correction, childhood anemia in Puno would only be 5.8%. Likewise, the region with the second highest percentage of anemia using WHO criteria would be the rainforest Loreto region, whereas, without the WHO cutoff of 11 g/dL, it would have the highest rate (61.5%). (Table 6) Residential altitude is a critical factor for the definition of anemia. The WHO tried to take the effect of altitude into account using an altitude-correction factor; however, it still overestimated anemia prevalence in high-altitude samples from Bolivia[13], Peru[17], and Ethiopia[19]. Using our four Hb curves related to altitude (Fig 2) and children's age in months, we could determine more precisely the prevalence of anemia. When we use our Hb percentiles stratified by age and altitude curves, the rainforest region has the highest anemia prevalence, and those living at high altitude, the lowest. There are eleven Peruvian regions with anemia values over the national average, including the five rainforest regions (Table 3 and Fig 3). The first three are along the Peruvian-Brazilian rainforest border. Considering the three Peruvian natural regions (highlands, rainforest, coast), anemia is more prevalent in the rainforest (13.5%) and reduced in frequency in the highlands (3.3%, p<0.0001, Table 7). Children living in the rainforest are more likely to be anemic because they have the lowest access to continuous public sewage and water services [76], a higher incidence of diarrheal diseases [77], a higher risk for malaria [78], greater low protein food consumption [79], an insufficiently nutrient-dense diet for children [80], less access to clean drinking water, and more often frequency to open defecation and soil-transmitted helminthes [81]. That is why it is misleading to use the WHO Hb cutoff values, which show no difference between highlands (52.4%) and rainforest (53.8%, p = 0.395) regions in anemia prevalence, and is opposite from what we found in our analysis. Specifically, our anemia cutoff curves for four different levels of altitude showed that living at low altitude had the highest anemia prevalence (8.8%) and those at high altitude the lowest (1.2%). Therefore, the 70.5% of anemia at very high altitudes as identified using the WHO criteria most likely represents an overestimation.

We propose that measures to increase the access to safe drinking water and to reduce chronic malnutrition and the use of traditional solid fuels stoves in the rainforest, could reduce the prevalence of anemia as much as 45%, 33% and 25%, respectively. (S2 Table) These measures are yet to be proposed in Peruvian national anemia campaigns.

The limitations of our analyses are similar to those of other studies trying to obtain population-specific Hb curves. First, the cutoff of the 5^th^ percentile is arbitrary. We used it because it was the basis of the 11 g/dL cutoff by WHO, and it has been used in the US and other countries for rate comparisons. Another limitation is that we only had access to Hb, and no other laboratory measures for defining anemia, similar to other studies [82].

In conclusion, we provide data to suggest that the anemia prevalence was 7.3% for Peruvian children 6 to 35 months old in 2016 and 2017. We obtained these data by performing a secondary analysis of the data obtained from the ENDES survey, taking into account the effects of age and altitude of residence. We have built four different Hb tables using the altitude of residence and age in order to be able to readily determine if a child is anemic or not. (Table 3)

## Supporting information

S1 Text Suplementary material. (DOCX)

S1 Table Estimated Hb summary statistics by year and the corresponding data available from those years. (DOCX)

S2 Table p5 anemia rates by natural region. (DOCX)

S3 Table WHO anemia rates by natural region. (DOCX)

S4 Table Percentage of people with the potential conditions affecting hemoglobin levels by anemia rates, WHO and p5 comparison. (DOCX)
