# Supplementary material for: Age and altitude of residence determine anemia prevalence in Peruvian 6 to 35 months old children
Source: PLoS One. 2020 Jan 15;15(1):e0226846. doi: 10.1371/journal.pone.0226846 (PMC6961872; doi:10.1371/journal.pone.0226846)
Supplement: S1 Text — (DOCX) [file pone.0226846.s001.docx]

The characteristics of Hb means in eutrophic and children with malnutrition are provided in S1 Table for the 2009-2013, 2012-2014 and 2015-2017 periods. This distinction obeys to the changes in ENDES sampling methodology over the years to reduce variability in health indicators (especially at sea level) (1).

DHS suggests that acceptable datasets produced using Hemocue®-based hemoglobinometers have a hemoglobin standard deviation between 1.1 to 1.5g/dL. (2) In Supplementary table 1 we show that early surveys have high SD values, which may indicate poor quality of data. This correlates with the relatively low sample taken compared against the final years. Differences in methodology across years were also taken into account for our decision to elaborate the paper utilizing the latest 2017 data.

**References**

1. Instituto Nacional de Estadística e Informática. Encuesta Demográfica y de Salud Familiar - ENDES 2017. Lima, Perú:INEI. 2018.
2. Pullum, Thomas, Deborah Kortso Collison, Sorrel Namaste, and Dean Garrett. 2017. Hemoglobin Data in DHS Surveys: Intrinsic Variation and Measurement Error. DHS Methodological Reports No. 18. Rockville, Maryland, USA: ICF.
