# Supplementary material for: Age and altitude of residence determine anemia prevalence in Peruvian 6 to 35 months old children
Source: PLoS One. 2020 Jan 15;15(1):e0226846. doi: 10.1371/journal.pone.0226846 (PMC6961872; doi:10.1371/journal.pone.0226846)
Supplement: S1 Table — (DOCX) [file pone.0226846.s002.docx]

**S1 Table.** **Estimated Hb summary statistics by year and the corresponding data available from those years**.

| Year | Mean Hb (g/dL) | SD | [Mean 95% CI] | | Total (n) | 6-35 months (n) | Healthy children (n) |
| --- | --- | --- | --- | --- | --- | --- | --- |
| 2017 | 11.68 | 1.41 | 11.64 | 11.72 | 22483 | 11578 | 6167 |
| 2016 | 11.67 | 1.42 | 11.63 | 11.71 | 22682 | 11530 | 5692 |
| 2015 | 11.70 | 1.45 | 11.66 | 11.74 | 25527 | 13169 | 6314 |
| 2014 | 11.61 | 1.48 | 11.55 | 11.67 | 10222 | 4997 | 2282 |
| 2013 | 11.65 | 1.51 | 11.58 | 11.72 | 9574 | 4726 | 2022 |
| 2012 | 11.78 | 1.53 | 11.71 | 11.85 | 10231 | 5100 | 2269 |
| 2011 | 11.90 | 1.56 | 11.83 | 11.97 | 9582 | 4848 | 1988 |
| 2010 | 11.68 | 1.53 | 11.61 | 11.76 | 9812 | 4906 | 1951 |
| 2009 | 11.68 | 1.53 | 11.61 | 11.76 | 10704 | 5260 | 1886 |

SD = Standard deviation, CI = Confidence interval.
