# Supplementary material for: Age and altitude of residence determine anemia prevalence in Peruvian 6 to 35 months old children
Source: PLoS One. 2020 Jan 15;15(1):e0226846. doi: 10.1371/journal.pone.0226846 (PMC6961872; doi:10.1371/journal.pone.0226846)
Supplement: S2 Table — (DOCX) [file pone.0226846.s003.docx]

**S2 Table.** **p5 anemia rates by natural region**.

|  | Solid fuels | | | | Measures to clean water | | | | Chronic malnutrition | | | |
| --- | --- | --- | --- | --- | --- | --- | --- | --- | --- | --- | --- | --- |
| Natural regions | No | n | Yes | n | No | n | Yes | n | No | n | Yes | n |
| Coast | 7.1%ᵃ | 3951 | 8.7%ᵇ | 708 | 10.4%ᵍ | 322 | 7.2%ʰ | 4383 | 7.4%ᵅ | 3907 | 7.4%ᵝ | 789 |
| Highlands | 3.3%ᶜ | 1526 | 3.2%ᵈ | 2113 | 4.0%^i^ | 241 | 3.2%ʲ | 3423 | 3.4%ᵞ | 2180 | 3.1%ᵟ | 1483 |
| Rain forest | 11.2%ᵉ | 1429 | 14.9%ᶠ | 1550 | **20.0%ᵏ** | 683 | **11.0%ˡ** | 2312 | **11.4%ᵋ** | **2051** | **17.1%ᶿ** | 943 |

Bonferroni-corrected linear combination of estimates p value: ᵃᵇ = 0.823, ᶜᵈ=0.805, **ᵉᶠ=** 0.065 ,ᵍ ʰ= 0.392, ⁱ ʲ=0.613 ,**ᵏ ˡ=<0.0001**, ᵅᵝ=0.997, ᵞᵟ=0.679, **ᵋᶿ=0.011**.
