# Supplementary material for: Age and altitude of residence determine anemia prevalence in Peruvian 6 to 35 months old children
Source: PLoS One. 2020 Jan 15;15(1):e0226846. doi: 10.1371/journal.pone.0226846 (PMC6961872; doi:10.1371/journal.pone.0226846)
Supplement: S3 Table — (DOCX) [file pone.0226846.s004.docx]

**S3 Table. WHO anemia rates by natural region.**

|  | Solid fuels | | | | Measures to clean water | | | | Chronic malnutrition | | | |
| --- | --- | --- | --- | --- | --- | --- | --- | --- | --- | --- | --- | --- |
| Natural regions | No | n | Yes | n | No | n | Yes | n | No | n | Yes | n |
| Coast | 34.9%ᵃ | 3951 | 43.3%ᵇ | 708 | 44.9%ᵍ | 322 | 35.6%ʰ | 4383 | 34.5%ᵅ | 3907 | 44.5%ᵝ | 789 |
| Highlands | 48.0%ᶜ | 1526 | 55.5%ᵈ | 2113 | 52.5%i | 241 | 52.4%ʲ | 3423 | 49.8%ᵞ | 2180 | 56.2%ᵟ | 1483 |
| Rain forest | 48.3%ᵉ | 1429 | 57.3%ᶠ | 1550 | 62.9%ᵏ | 683 | 50.3%ˡ | 2312 | 51.5%ᵋ | 2051 | 57.9%ᶿ | 943 |

Bonferroni-corrected Linear combination of estimates p value: ᵃᵇ = <0.0001,ᶜᵈ= <0.0001,ᵉᶠ= <0.0001 ,ᵍ ʰ= 0.039, ⁱ ʲ= 0.988,ᵏ ˡ= <0.0001, ᵅᵝ=<0.0001, ᵞᵟ=0.006, ᵋᶿ=0.039.
