# Supplementary material for: Age and altitude of residence determine anemia prevalence in Peruvian 6 to 35 months old children
Source: PLoS One. 2020 Jan 15;15(1):e0226846. doi: 10.1371/journal.pone.0226846 (PMC6961872; doi:10.1371/journal.pone.0226846)
Supplement: S4 Table — (DOCX) [file pone.0226846.s005.docx]

**S4 Table. Percentage of people with the potential conditions affecting hemoglobin levels by anemia rates, WHO and p5 comparison.**

|  |  | Measures to clean water (yes, %) | n (group) | Exposure to solid fuels (yes, %) | n (group) | Chronic malnutrition (Yes, %) | n (group) |
| --- | --- | --- | --- | --- | --- | --- | --- |
| Anemia % (p5) | Yes | **80.88%** | 858 | 38.02% | 851 | 29.20% | 857 |
|  | No | **90.00%** | 10118 | 34.50% | 4371 | 27.07% | 3215 |
|  | p | **<0.0001** | | **<0.0001** | | 0.3414 | |
| Anemia % (WHO) | Yes | 86.70% | 5292 | 42.30% | 5247 | 32.60% | 5290 |
|  | No | 91.50% | 6072 | 28.90% | 6030 | 23.00% | 6063 |
|  | p | **<0.0001** | | **<0.0001** | | **<0.0001** | |
